# Omega-3 Fatty Acids and Eye Health: Opinions and Self-Reported Practice Behaviors of Optometrists in Australia and New Zealand

**DOI:** 10.3390/nu12041179

**Published:** 2020-04-22

**Authors:** Alexis Ceecee Zhang, Sumeer Singh, Jennifer P. Craig, Laura E. Downie

**Affiliations:** 1 Department of Optometry and Vision Sciences, The University of Melbourne, Carlton, VIC 3053, Australia; alexisz@student.unimelb.edu.au (A.C.Z.); shari@student.unimelb.edu.au (S.S.); 2 Department of Ophthalmology, New Zealand National Eye Centre, The University of Auckland, Auckland 1023, New Zealand; jp.craig@auckland.ac.nz

**Keywords:** omega-3, fatty acid, diet, supplement, optometrist, survey, nutrition, practice, eye disease, dry eye, age-related macular degeneration

## Abstract

This study investigated optometrists’ attitudes and self-reported practice behaviors towards omega-3 fatty acids for eye health, and knowledge and understanding of their potential risks and benefits. An anonymous online survey was distributed to optometrists in Australia and New Zealand. Questions included practitioner demographics and practice modality; self-reported practices and recommendations relating to diet, nutritional supplements, and omega-3 fatty acids for age-related macular degeneration (AMD) and dry eye disease (DED); and practitioner knowledge about omega-3 fatty acids. Of 206 included surveys, most respondents (79%) indicated recommending for their patients to consume omega-3 fatty acids to improve their eye health. Sixty-eight percent of respondents indicated recommending omega-3-rich foods for AMD management, while 62% indicated recommending omega-3 supplements. Most respondents (78%) indicated recommending omega-3-rich foods or supplements for DED. For DED, recommended omega-3 supplement dosages were (median [inter-quartile range, IQR]) 2000 mg [1000–2750 mg] per day. The main sources of information reported by respondents to guide their clinical decision making were continuing education articles and conferences. In conclusion, optometrists routinely make clinical recommendations about diet and omega-3 fatty acids. Future education could target improving optometrists’ knowledge of differences in the evidence for whole-food versus supplement sources of omega-3 fatty acids in AMD. Further research is needed to address uncertainties in the evidence regarding optimal omega-3 dosage and formulation composition in DED.

## 1. Introduction

Diet is a major lifestyle factor that can influence eye health [1]. There is evidence to suggest that diets rich in omega-3 essential fatty acids, obtained from food sources or supplementation, may have ocular benefits. Omega-3 fatty acids are termed ‘essential’ as they cannot be synthesized in the body and, thus, must be obtained from the diet. The other major essential fatty acid family is the omega-6 fatty acids. A person’s dietary intake and balance of omega-6 and omega-3 fatty acids has been reported to be important for regulating vascular activity, mediating immune and nervous system function, and influencing the balance of systemic lipid-derived mediators [2].

Omega-3 fatty acids can be obtained from several sources. Short-chain omega-3, alpha-linoleic acid (ALA), is found mostly in plant-based foods (e.g., flaxseed, chia seeds). Long-chain omega-3 fatty acids, docosahexaenoic acid (DHA) and eicosapentaenoic acid (EPA), are predominantly obtained from marine-based foods (e.g., oily fish). As the biological conversion of ALA to EPA and DHA is incomplete in vivo (estimated in the range of 5 to 20% conversion) [3,4,5], direct intake of long-chain omega-3 fatty acids is considered preferable [6]. Once ingested, long-chain omega-3 fatty acids compete with the long-chain omega-6 fatty acid, arachidonic acid (AA), for incorporation into cellular membranes [7]. Countering pro-inflammatory AA-mediated pathways, increasing omega-3 fatty acid intake modulates systemic inflammation through the production of anti-inflammatory metabolites. The optimal ratio of dietary omega-6 to omega-3 is considered to be approximately 4 to 1 [8]. Excessive consumption of foods rich in omega-6 fatty acids, a recognized feature of modern Western diets, can shift the omega-6:omega-3 ratio closer to 15 to 1 [8]. Moreover, it has been reported that up to 80% of adults in developed countries may not obtain the daily, recommended dietary intake of long-chain omega-3 fatty acids for optimal health [9]. Diets rich in long-chain omega-3 fatty acids have been suggested to provide long-term benefits for several chronic ocular conditions, including dry eye disease (DED) and age-related macular degenerations (AMD) [10,11].

DED is one of the most common reasons for patients to seek ophthalmic care [12]. DED can impose a significant burden on quality of life and is associated with substantial direct and indirect health costs [13,14]. The condition is characterized by a loss of tear film homeostasis and potential disruption to ocular surface integrity [15]. DED is commonly associated with ocular surface inflammation [16]. Dietary modification with omega-3 fatty acids is thought to reduce DED symptoms and signs by modulating ocular surface inflammation and improving tear-lipid profiles [17,18]. The United States (US) Women’s Health Study showed a higher incidence of DED in women with low dietary omega-3 fatty acid intake [19]. However, clinical trials assessing the potential effects of omega-3 supplementation on the clinical expression of DED have presented apparently contradictory findings [20,21,22,23,24]. A recent Cochrane systematic review concluded that while a possible role exists for long-chain omega-3 supplements in managing DED, the best available evidence is currently uncertain and inconsistent [25]. The role of omega-3 fatty acids in the management of DED is, thus, of continued interest and debate.

In addition to its anti-inflammatory effects, DHA is implicated in maintaining the structural and functional properties of the retina [26]. Diets rich in polyunsaturated fatty acids improve the retinal cellular response to ischemic, oxidative, and inflammatory damage in animal models of AMD [27,28,29]. Epidemiology studies have associated dietary long-chain omega-3 intake with a lower risk of developing early-stage AMD [30] and progression to late-stage, sight-threatening forms of disease [31]. However, omega-3 nutritional supplements have been shown not to confer the same benefit of reducing the risk of developing AMD as whole foods [32]. The reason for this difference in efficacy may be potential synergistic interactions of fatty acids with other nutrients and vitamins that are present in whole foods but not supplements [33]; these differences should be recognized when clinical recommendations relating to omega-3 fatty acids are made for AMD. 

Eye care clinicians have an important role in informing their patients and the public about modifiable risk factors for ocular disease, including diet. Optometrists, as major providers of primary eye care in Australia and New Zealand, are ideally positioned to counsel patients about the benefits and risks of nutritional supplements and their potential impact on eye health. Two studies have investigated the self-reported recommendations made by optometrists relating to nutrition. In the UK, Lawrenson and Evans (2013) reported active engagement by optometrists in providing nutritional advice relating to AMD, but highlighted a need for increased awareness of the relevant research evidence [34]. In an Australian study of self-reported optometric practice patterns, Downie and Keller (2015) found 60% of optometrists indicated routinely asking their patients about diet, and approximately half indicated asking about nutritional supplement intake [35]. To date, no studies have specifically investigated eye care practitioner recommendations relating to omega-3 fatty acids.

In both Australia and New Zealand, the professional scope of optometry includes the diagnosis, assessment, and management of ocular disorders, including the prescription of topical and, in New Zealand, oral medications. In both countries, optometrists are assessed to equivalent competency standards [36]. Research has highlighted that most of the general public who seek optometric care expect to be counselled about their diet, as it relates to their eye health [37]. It is, thus, critical that the information delivered by eye care providers is accurate and up to date.

The aim of this study was to investigate the current attitudes and self-reported practice behaviors of optometrists towards omega-3 fatty acid recommendations for eye health, and to assess their opinions and understanding of the potential benefits and risks associated with oral omega-3 fatty acid intake. 

## 2. Materials and Methods 

### 2.1. Participants

An anonymous, web-based survey was distributed to optometrists in Australia and New Zealand from June to October 2019. The survey was circulated electronically through professional organizations (Optometry Australia and associated early career networks, Cornea and Contact Lens Society of Australia, New Zealand Association of Optometrists, and Cornea and Contact Lens Society, New Zealand) and at a major national professional education conference, held in Melbourne Australia (July 2019). 

The project was approved by both the University of Melbourne Human Research Ethics Committee (HREC #1853287.1) and University of Auckland Human Participants Ethics Committee on 12 March 2019 for three years (ref. 022730).

### 2.2. Survey Design

This survey was administered using Qualtrics (Qualtrics, Provo, UT, USA) and comprised 39 questions, divided into four sections. Questions within each section sought responses that included: Select yes/no, select all that apply, select one option only, and optional free-text boxes. Reviewing or altering prior responses was not permitted. The survey was piloted to optimize the clarity of questions and the flow. Respondents were informed at the beginning of Section 2 that, for the purpose of this survey, diet was defined as “the food(s) and drink(s) that are regularly consumed”. 

**Figure 1:**
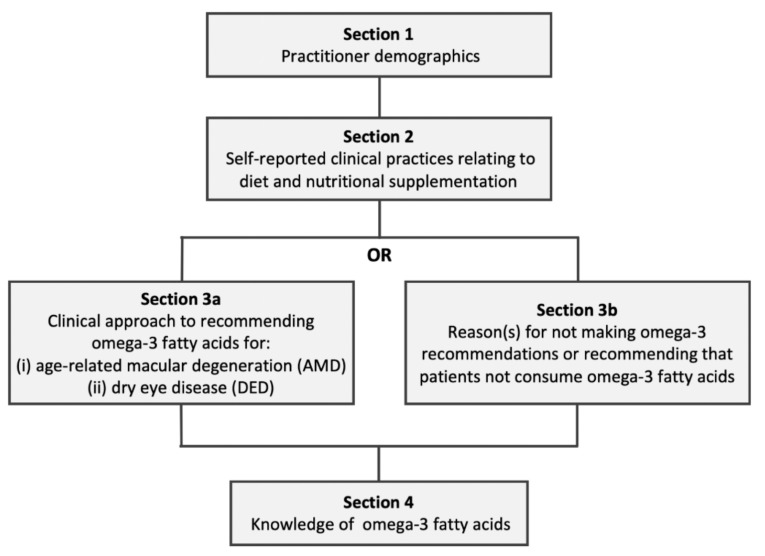
Survey structure.

Figure 1 illustrates the survey flow and question logic. As summarized in Table 1, the survey’s four sections comprised: (1) Participant demographics, (2) self-reported clinical practices relating to diet and nutritional supplementation, (3a) clinical approach to recommending omega-3 fatty acids for AMD and DED, or (3b) reason(s) for not making omega-3 recommendations or for recommending that patients not consume omega-3 fatty acids, (4) knowledge relating to omega-3 fatty acids. 

After completing Section 2, branch logic was used to direct respondents to either Section 3a or Section 3b depending on their current general clinical approach to omega-3 fatty acids, according to their response to the following three options:

(1) I make recommendations for my patients to consume omega-3 fatty acids (in either oral supplementation form or via dietary modification) to improve their ocular health;
(2) I do not make recommendations for my patients to consume omega-3 fatty acids (in either oral supplementation form or via dietary modification) to improve their ocular health;
(3) I recommend for my patients not to consume omega-3 fatty acids to improve their ocular health.

Participants who selected option (1) were directed to complete Section 3a (to obtain data about their clinical recommendations relating to omega-3 fatty acids for AMD and DED). Participants who selected options (2) or (3) were directed to complete Section 3b (to explore reasons for not making clinical recommendations relating to omega-3, or recommending that patients not consume omega-3 fatty acids).

### 2.3. Data Analysis

Data contributed by optometrists practicing outside of Australia or New Zealand, or from participants who did not complete the survey up to the end of Section 3, were not included in the analysis.

Statistical analysis was performed using IBM SPSS statistics software version 21.0 (https://www.ibm.com/analytics/spss-statistics-software). Graphs were plotted in GraphPad PRISM (version 6.01 for Windows, GraphPad Software, La Jolla, CA, USA, www.graphpad.com). Descriptive statistics were used to analyze participant demographics, clinical practices relating to diet and nutrition, clinical practice relating to omega-3 fatty acids, and practitioner knowledge of omega-3 fatty acids. Fisher’s exact test was used to compare data relating to the proportions of respondents across different groups. Specific recommendations relating to omega-3 supplementation, derived from responses in the free-text boxes, were represented using a ‘word cloud’ (Wordle^TM^; http://www.wordle.net/). The size of the lettering in the ‘word cloud’ reflects the frequency with which respondents reported the individual words.

Univariate and multivariate binocular logistic regression analyses were performed to assess factors influencing a clinician’s self-reported practices relating to omega-3 fatty acid recommendation for ocular health, and knowledge relating to the biological effects and dietary targets of omega-3 fatty acids. A p-value of less than 0.05 was used to define statistical significance. To quantify participant knowledge about omega-3 fatty acids, the accuracy of respondents’ answers was scored out of five (i.e., one point for each correctly answered question). The five knowledge questions surveyed and the answers that were considered as ‘correct’ are provided in Table A1. A score of three or more (i.e., 60%) was considered a ‘pass’ for the ‘omega-3 knowledge score’.

## 3. Results

### 3.1. Participant Demographics

Of 234 total responses received, 206 responses were included in the analysis. Twenty-eight surveys were excluded because they were either not completed up to the end of Section 3 or were completed by optometrists practicing outside of Australia and New Zealand.

Table 2 summarizes the demographics of participating optometrists. Approximately two-thirds of responses were from optometrists practicing in Australia (64%) and one-third practicing in New Zealand (36%). The majority of respondents were endorsed to prescribe ocular therapeutic medications (Australia, 86%; New Zealand, 91%). 

Most respondents completed their optometry training in Australia or New Zealand (94%); the remainder completed their degree in the UK, South Africa, or US (and additional competency examinations to practice optometry in Australia or New Zealand). Australian respondents practiced in all 6 states; most were practicing in Victoria (48%), New South Wales (25%), and Queensland (12%). From New Zealand, responses were received from optometrists in all 16 regions except Gisborne, Tasman, Marlborough, West Coast, and Southland. Most New Zealand optometrists practiced in Auckland (35%), Waikato (14%), Wellington (13%), and Hawkes Bay (13%). 

Approximately half of the respondents worked in independent optometric practice (49%), 30% worked principally in corporate practice, 11% in a hospital or public health clinic, and 8% within academia. The vast majority of respondents (92%) selected one or more areas of clinical interest, with 50% self-declaring interest in DED or ocular surface disease, 26% in retinal disease, and 8% in gerontology or aged care.

### 3.2. Clinical Recommendations Relating to Diet

**Figure 2:**
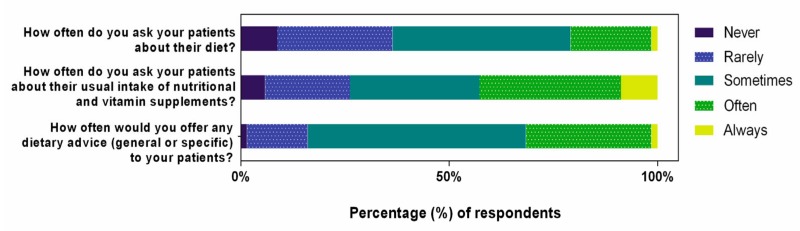
Optometrists’ self-reported clinical practices as related to diet and nutritional supplementation. Percentage (%) of respondents, from *n =* 206, who selected each frequency of practice, on a five-step Likert scale.

As responses from Australian and New Zealand practitioners were similar, pooled data across both locations are presented. Figure 2 summarizes self-reported clinical practice behaviors relating to diet and nutritional supplements.

Of the 91% of respondents who asked their patients about their diet, 98% did not use any quantitative tools to survey dietary habits. Three respondents indicated that they used either a food frequency questionnaire (*n =* 2) or an online dietary intake tool (*n =* 1). Optometrists reported more frequently (i.e., often or always) asking their patients about their nutritional supplement intake than about their diet (43% versus 21%; odds ratio (OR): 2.83, 95% confidence interval, CI: 1.83 to 4.37; *p <* 0.001). Approximately one in three optometrists reported often or always (32%), and one in two, sometimes (52%) providing patients with general or specific dietary advice. 

Most respondents (79%) indicated that they recommend the consumption of omega-3 fatty acids to patients, in either supplement form or from food sources, to improve eye health. The remaining (21%) respondents indicated that they did not make any recommendations to their patients regarding omega-3 fatty acids. None of the respondents recommended that their patients avoid consuming omega-3 fatty acids.

For the 44 respondents who reported not making any recommendations relating to omega-3 fatty acids, almost half (47%) indicated that they felt like they did not know enough about omega-3 fatty acids to justify making recommendations. Thirteen respondents felt there was insufficient published research evidence to support omega-3 fatty acid recommendation for eye health, and three indicated that the published evidence they were aware of showed that omega-3 fatty acids were not beneficial for ocular health. Seven respondents either themselves considered or perceived that their patients considered nutritional advice to be outside the scope of optometric practice. 

Four respondents indicated that they previously recommended omega-3 fatty acids to their patients, but were no longer doing so. Reason(s) provided included that: they did not see any clinical benefits in their own patients (*n =* 1), recent evidence had changed their opinions (*n =* 1), or they would recommend other forms of therapy (e.g., intense pulsed light therapy for DED) over dietary interventions (*n =* 2). Of the 44 respondents who did not make any recommendations relating to omega-3 fatty acids, 20% (*n =* 9) indicated recommending supplement formulations based on the results of the Age-Related Eye Disease Studies (AREDS and AREDS II) for their patients with AMD.

Table 3 summarizes the factors that predicted whether optometrists recommended omega-3 fatty acid consumption to patients to improve their ocular health, from either dietary modification or oral supplementation. In univariate analysis, optometrists were more likely to recommend omega-3 fatty acids if they worked in an independent practice (OR: 4.06, 95% CI: 1.26–13.06; *p =* 0.019), had a self-declared clinical interest in DED or ocular surface disease (OR: 4.58, 95% CI: 2.12–9.91; *p <* 0.001), or if they recognized that omega-3 fatty acid supplements had potential side effects (OR: 3.94, 95% CI: 1.88–8.25; *p <* 0.001). In multivariate analysis, both a clinical interest in DED or ocular surface disease (OR: 3.10, 95% CI: 1.31–7.36; *p =* 0.010) and recognition that omega-3 fatty acids have side effects (OR: 2.34, 95% CI: 1.05–5.22; *p =* 0.037) were significant factors in predicting whether omega-3 fatty acids were recommended to improve ocular health. 

### 3.3. Recommendations Relating to Omega-3 Fatty Acids in Age-Related Macular Degeneration (AMD) 

**Figure 3:**
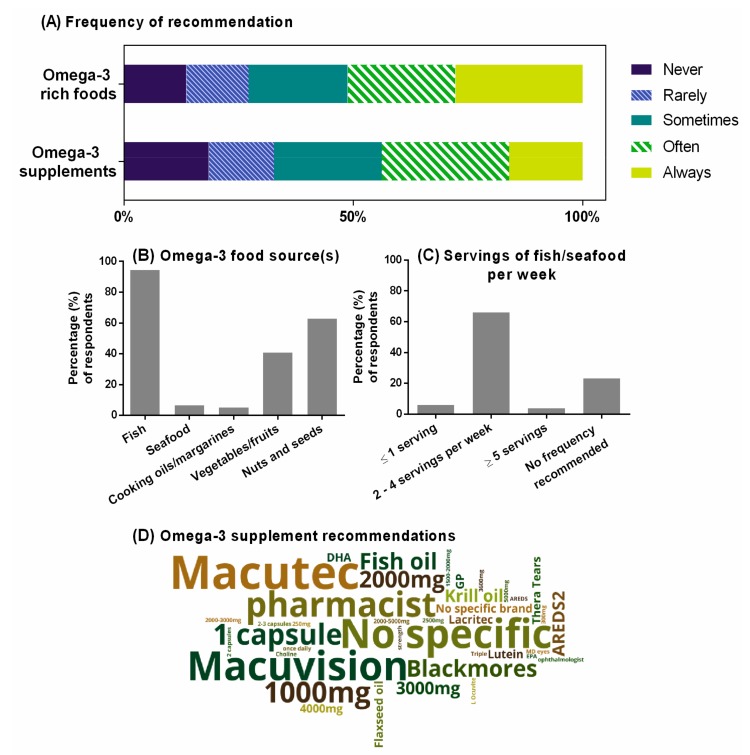
Optometrists’ self-reported clinical practices as related to omega-3 fatty acid intake as a component of AMD management. (**A**) Self-reported frequency of recommending omega-3-rich foods and nutritional supplements. Percentage of respondents shown from *n =* 162 who indicated recommending omega-3 fatty acids for AMD. (**B**) Omega-3 food sources(s) recommended by respondents. Percentage shown from *n =* 140 who indicated recommending omega-3-rich foods. (**C**) Recommended ideal frequency for patients to consume marine-based (long-chain) omega-3-containing foods. Percentage shown from *n =* 133 who indicated recommending fish or seafood as omega-3 food sources in (**B**). (**D**) Word-cloud representation of specific recommendations for omega-3 supplementation (based on *n =* 80 responses from the 132 participants who indicated recommending omega-3 supplements).

Figure 3A summarizes the self-reported practice behaviors of optometrists relating to omega-3 fatty acid intake, through either diet or supplementation, as a component of clinical management for people with AMD. Overall, 68% of optometrists indicated recommending omega-3 rich foods for AMD (Figure 3B). Almost all (95%) recommended fish or non-fish seafood as a food source. Almost 80% of optometrists who recommended fish or seafood specified the preferred frequency of consumption, and this was most commonly two to four servings per week (Figure 3C). Two-thirds of optometrists who recommended omega-3 rich foods for AMD suggested nuts and seeds (63%), and 40%, vegetables or fruits.

Participants who indicated recommending omega-3 supplements were invited to detail the specific recommendation(s) given to their patients (such as dose, frequency, brand). Figure 3D provides a visual representation of these free-text responses received from 80 of the 132 practitioners (61%), constructed using a ‘word cloud’. Supplements broadly based on the formulation in the AREDS studies [38] (Macutec (Stiltec Pty Ltd, Queensland, Australia), Macuvision (Blackmores, New South Wales, Australia), or MD Eyes (MD EyeCare, Queensland, Australia)) were listed by 21% of optometrists who provided free-text responses (*n =* 17). About one-third of respondents (29%) provided specific dosages in their recommendations (*n =* 23), and these ranged from 250 mg to 5000 mg of omega-3 fatty acids per day (Median [Inter-quartile range, IQR]: 2000 [1000–3000] mg/day). Twelve percent of respondents (*n =* 10) indicated that they do not make specific recommendations relating to the brand or dosage of omega-3 supplement, and 11% percent indicated that they would advise their patients to consult either a pharmacist or a general practitioner for advice relating to specific supplementation products and the dosage.

### 3.4. Recommendations Relating to Omega-3 Fatty Acids in Dry Eye Disease (DED)

**Figure 4:**
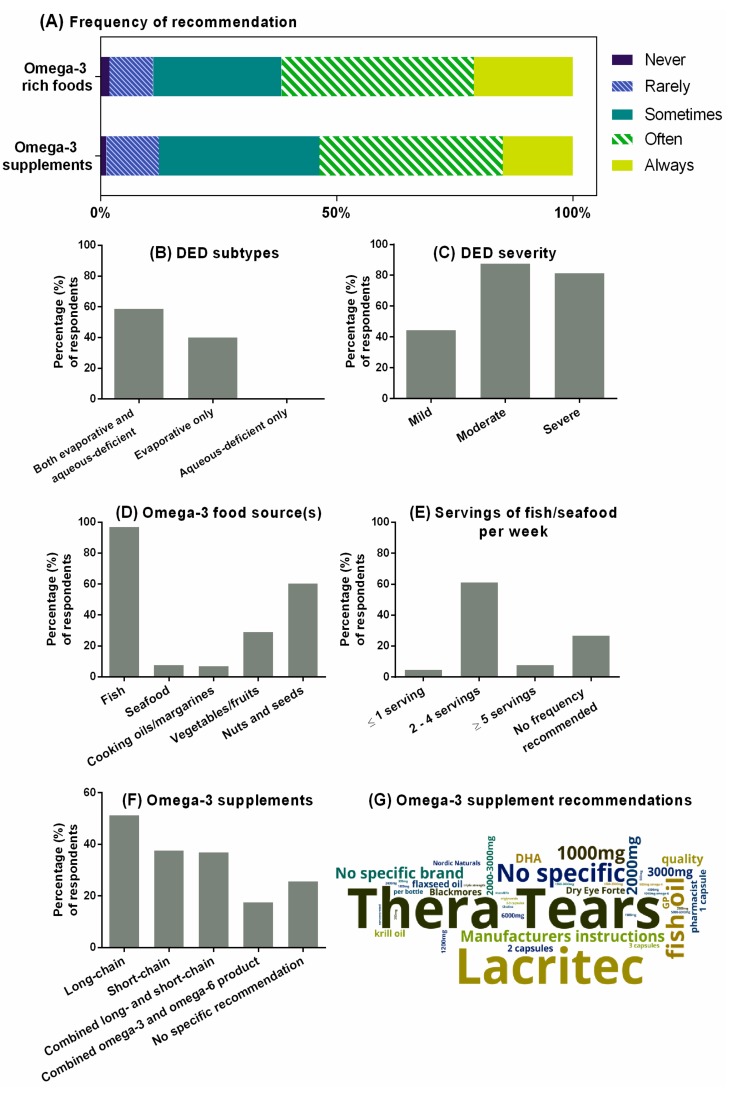
Optometrists’ self-reported clinical practices as related to omega-3 fatty acids for managing DED. (**A**) Self-reported frequency of recommending omega-3-rich foods and nutritional supplements. Percentage of respondents shown from *n =* 162 who indicated recommending omega-3 fatty acids for DED, (**B**) DED subtypes, and (**C**) severities that participants self-reported making recommendations for omega-3 fatty acids (in either food or supplement forms). Percentages shown from *n =* 162. (**D**) Type of omega-3 food source(s) recommended. Percentage shown from *n =* 159 who indicated recommending omega-3-rich foods. (**E**) Recommended ideal frequency for patients to consume marine-based (long-chain) omega-3-rich foods. Percentages shown from *n =* 154 who indicated recommending fish or seafood as omega-3 food sources in (e). (**F**) Type of oral omega-3 supplements recommended. Percentages shown from *n =* 160 who indicated recommending omega-3 supplements. (**G**) Word-cloud representation of specific recommendations for omega-3 supplementation (based on *n =* 105 responses from the 160 participants who indicated recommending omega-3 supplementation).

Figure 4A summarizes the self-reported practice behaviors of optometrists who recommended omega-3 fatty acids as a component of DED management. Compared with AMD (68%), a higher proportion of respondents (78%) indicated recommending omega-3 fatty acids from food sources or supplements to manage DED. Almost all (>99%) of respondents who made this recommendation considered omega-3 fatty acids to be appropriate for evaporative and/or mixed etiologies of DED (Figure 4B). Only one practitioner indicated recommending omega-3 fatty acids specifically for the aqueous-deficient DED subtype. Practitioners were less likely to recommend omega-3 fatty acids to patients with mild, compared to moderate or severe forms of, DED (Figure 4C, OR: 0.24, 95% CI: 0.16 to 0.37; *p <* 0.001).

Self-reported clinical recommendations relating to dietary sources of omega-3-rich foods and frequency of intake in DED were similar to those for AMD (Figure 4D). Of the 77% of optometrists who recommended for patients to increase their intake of foods rich in omega-3 fatty acids to improve their DED, almost all (97%) specified either fish or non-fish seafood sources, most commonly between two and four servings per week (Figure 4E). Over half of respondents recommended nuts and seeds (60%), and one in three indicated value in consuming vegetables or fruits to obtain omega-3 fatty acids. 

Oral omega-3 supplements were recommended by 78% of optometrists for managing DED (Figure 4F). The most frequently recommended forms were long-chain omega-3 fatty acids from marine-based sources (40% of all optometrists), short-chain omega-3 from plant-based sources (29% of all optometrists), and combined long- and short-chain omega-3 fatty acids (29% of all optometrists). Omega-3 and omega-6 combination products were recommended by 14% of optometrists for DED.

Figure 4G shows a ‘word cloud’ representation of the free-text responses from the 105 of 160 practitioners (65%) who provided specific recommendations regarding omega-3 supplements for DED. As represented by the size of the lettering, Thera Tears (Akorn Consumer Health, MI, USA) and Lacritec (Stiltec Pty Ltd, QLD, Australia) were the two most frequently recommended commercial brands, listed by 17% and 15% of respondents, respectively. About one-third of respondents provided specific omega-3 dosage recommendations for DED (31%), which ranged from 250 mg to 6000 mg per day (median [IQR]: 2000 [1000–2750] mg per day), and 10% (*n =* 10) of respondents advised their patients to consult either the instructions on the supplement bottle or the advice of a pharmacist. 

### 3.5. Practitioner Knowledge of Omega-3 Fatty Acids

Half of the respondents (50%) were not aware of a difference between the biological effects of plant-based (short-chain) and marine-based (long-chain) omega-3 fatty acids. About one-third (31%) correctly considered plant-based omega-3 fatty acids to have less, and 12% considered plant-based omega-3 fatty acids to have greater, biological effect than marine-based omega-3 fatty acids when consumed at the same dose. Just over half (51%) of respondents considered omega-3 fatty acids to be mostly anti-inflammatory, 15% of optometrists recognized that omega-3 fatty acids are always anti-inflammatory, and 42% of optometrists correctly considered omega-6 fatty acids to be mostly pro-inflammatory. The perceived ideal ratio of omega-6 to omega-3 in the human diet was most frequently selected as 1 to 4 (27% of optometrists), 16% of respondents correctly selected the ideal ratio to be 4 to 1, and 39% of optometrists were unsure of the ideal ratio. 

From a forced-choice list of five options ranging from 150 mg/day to 1500 mg/day, 65% of respondents nominated a daily recommended dietary target of long-chain omega-3 fatty acids (the remaining respondents selected ‘don’t know’). The most common daily targets selected were 1000 mg per day (24% of optometrists) and 1500 mg per day (17% of optometrists). One in 11 respondents correctly selected the ideal approximate target of 500 mg/day for adults. About half of respondents (52%) considered there to be a safety limit for daily omega-3 fatty acid consumption; the perceived limit selected by respondents encompassed a 20-fold dose range. From the four multiple choice options offered, 500 mg per day was considered the upper limit by 4% of all optometrists, 1500 mg per day by 15% of optometrists, 3000 mg per day by 18% of optometrists, and 5000 mg per day by 8% of optometrists. 

Over half (53%) of respondents believed that omega-3 fatty acids have potential side effects. Based on multiple-choice options (see Table 1), these were most commonly believed to be gastric reflux (37%), nose bleeds (23%), and bloating (11%). Forty-six percent of respondents considered possible contraindications to use before recommending omega-3 fatty acids. In open-text boxes, the most commonly suggested contraindications were concurrent anticoagulant medications (26%), allergies to fish/seafood (12%), or a scheduled surgical procedure (3%). Five percent of respondents indicated they would ask the patient to consult their general practitioner or specialist prior to commencing oral omega-3 supplementation. 

The level of respondents’ knowledge of omega-3 fatty acids was assessed using an ‘omega-3 knowledge score’, derived from five knowledge-specific questions in the survey (see Table A1). A ‘pass’ was considered a score of three or more out of five (i.e., at least 60%). Using this criterion, in both univariate and multivariate analysis (Table 4), optometrists with a self-reported clinical interest in DED or ocular surface disease were more knowledgeable about omega-3 fatty acids (OR: 2.97, 95% CI: 1.09 to 8.11; *p =* 0.034) relative to those without such interests. Compared with respondents working in academia, those who were based principally in a corporate practice setting had a lower omega-3 knowledge score (OR: 0.080, 95% CI: 0.008 to 0.086; *p =* 0.037).

### 3.6. Sources of Evidence and Information

**Figure 5:**
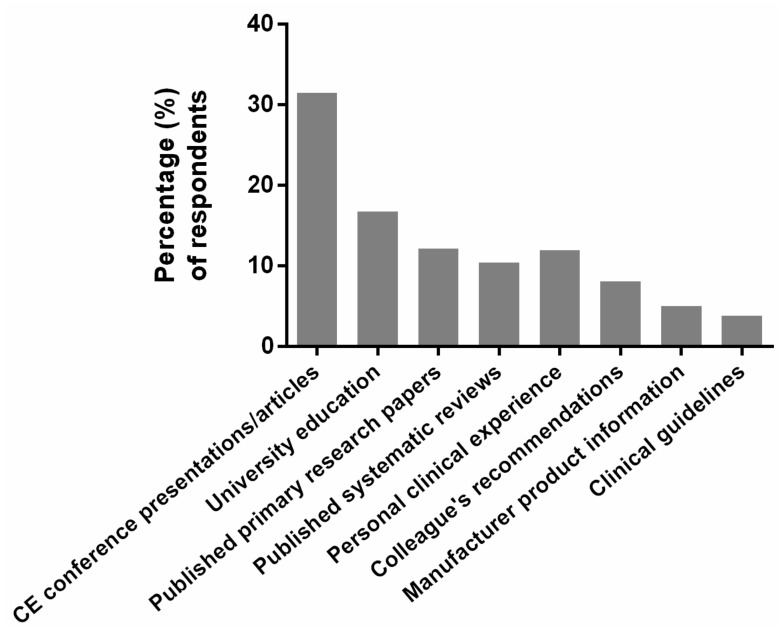
Percentage of optometrists who selected each information and/or evidence source (with no limit on the number of sources able to be selected) as informing their clinical decision-making regarding omega-3 fatty acids. CE, continuing education.

Figure 5 summarizes the sources of evidence and information that respondents indicated had informed their current clinical decision-making relating to omega-3 fatty acids. Continuing education conference presentations and articles were the major information sources followed by respondents’ university education and personal clinical experiences. Two in five optometrists (40%) indicated using published primary research papers and/or systematic reviews to guide their clinical decision-making in this area of practice.

## 4. Discussion

This study describes the self-reported clinical practices and opinions of Australian and New Zealand optometrists, as related to omega-3 fatty acids and eye health. To our knowledge, this is the first study to consider clinicians’ awareness and routine practices relating to omega-3 fatty acids in primary health care. The chosen focus on omega-3 fatty acids for this survey was based on their reported potential to improve outcomes in certain chronic ocular conditions, such as DED and AMD. The current findings provide new insights into optometrists’ understanding of current research evidence relating to omega-3 fatty acids in the context of eye health, and their self-reported application of this knowledge in practice. Furthermore, this survey considered the sources of information and evidence used to guide optometrists’ clinical decision-making as a foundation for informing the optimal mode(s) of delivering future education programs relating to nutrition in eye care practice. 

### 4.1. General Recommendations of Optometrists Relating to Diet and Nutrition

This survey, which was made available to an estimated 6500 practicing optometrists via professional optometry networks in Australia and New Zealand, received responses that were considered representative of the major regions in both countries. About half of respondents described their principal practice setting as independent (49%), which indicates a possible over-representation of responses received from independent practices compared to corporate or public health practices (local healthcare workforce data suggest ~30% optometry registrants practice in independent-settings practice [39]). Globally, there is substantial variation in optometric scope of practice. In some countries, such as France and Japan, optometrists are restricted to providing optical corrections using spectacles and/or contact lenses [40,41]. In contrast, in some states of the US and Canada, optometric practice includes performing refractive surgery procedures and minor ocular surgery [42,43,44]. The role of optometrists in Australia and New Zealand includes the independent diagnosis and management of ocular disorders relating to visual function and eye health [36,43]. In addition, optometrists who hold therapeutic medicine endorsement are permitted to prescribe topical, and in New Zealand oral, medications for managing eye disease. Overall, most survey respondents were qualified to prescribe topical ophthalmic therapeutic medications at a minimum (Australia, 86%; New Zealand, 91%). These proportions are higher than the documented percentage of therapeutically endorsed practitioners in both countries (Australia ~63%; New Zealand ~75%) [43,45]; this may be due to a self-selection bias based on clinical skill or interest, and/or that the survey responses were generally from a younger cohort of optometrists (44% under 30 years of age, compared with ~26% of all Australian optometrists being under the age of 30 [45]). Since 2013, all entry-level optometrists from accredited programs in Australia and New Zealand have graduated with therapeutic prescribing rights [36].

Optometrists, as major providers of primary eye care, are ideally placed to ask patients about their diet and to offer evidence-based advice about nutrition-based strategies for reducing the risk and/or progression of ocular disease. In a previous survey-based study, it was reported that two in five patients attending for optometric care in Australia expected their optometrists to ask them about their diet and/or nutritional supplement intake, although only one in five recalled being asked about these factors when attending an optometric consultation [37]. In the present study, most (64%) respondents self-reported generally (i.e., sometimes, often, or always) enquiring about their patients’ diet and/or their routine intake of nutritional or vitamin supplements (74%). These values are similar to findings reported in a cohort survey undertaken five years ago, which found that 62% of Australian optometrists self-reported counseling their patients about their diet, and 55% self-reported providing advice about nutritional supplementation [35]. 

### 4.2. Recommendations Relating to Omega-3 Fatty Acids for Managing AMD

Over 50% of respondents indicated that they would frequently recommend for their AMD patients to increase their intake of omega-3 rich foods. This is comparable to a previous survey, conducted in the UK, which found that about half of eye care professionals (optometrists or ophthalmologists) reported frequently recommending eating oily fish at least twice per week to individuals with AMD and/or individuals considered to be at risk of AMD [34]. 

Several prospective cohort studies have associated higher intakes of foods containing long-chain omega-3 fatty acids with a reduced risk of AMD progression [31,46]. The Blue Mountains Eye Study found that eating oily fish at least once per week, compared with less than once per week, was associated with a lower risk of developing early-stage AMD [47]. Systematic reviews, synthesizing results from primary research studies, have also found that eating approximately two servings of oily fish per week is associated with a reduced risk of both developing early AMD and progressing to late-stage disease [48,49]. Most optometrists who recommended consumption of fish or seafood, recommended an intake frequency of two or more servings per week, consistent with current evidence [48].

With respect to omega-3 fatty acid supplementation, 62% of respondents reported recommending either plant- or marine-based supplements for AMD management. Although it might be argued that a person’s diet can be augmented through supplementation if adequate levels of certain nutrients are not being obtained from food sources, the current best-available evidence does not support omega-3 oral supplements for preventing or slowing the progression of AMD [32]. In contrast to many epidemiological studies on diet, a Cochrane systematic review, which combined findings from two randomized controlled trials, reported that long-chain omega-3 supplementation for a period of up to five years did not reduce the development of vision loss or the risk of progression to advanced AMD [32]. Furthermore, in the AREDS II trial, the addition of xanthophyll carotenoids (lutein and zeaxanthin) and omega-3 fatty acids to the original high-dose antioxidant AREDS formulation did not further reduce the risk of progression to late-stage AMD relative to the original formulation [50]. This finding is in contrast to observational evidence that has reported the potential benefit of high dietary intakes of lutein and zeaxanthin for lowering the overall risk of developing late-stage AMD [51].

The absorption of EPA and DHA from nutritional supplementation is influenced by the formulation, chemical preparation, and a person’s background dietary fats [52,53]. Phospholipid and triglyceride forms are considered to have superior bioavailability compared to ethyl ester forms [53]. Nonetheless, nutritional supplements do not contain the full spectrum of nutrients present in whole foods. Consuming a supplement, rather than whole foods, may limit key interactions between the fatty acids and other nutrient components that could contribute towards the retinoprotective effect evident with omega-3 rich diets [54]. In the context of AMD, omega-3 supplements are, thus, not an equivalent substitute for omega-3 fatty acids from dietary sources. Our findings suggest that many optometrists may not be aware of this subtle, but important, distinction.

### 4.3. Recommendations Relating to Omega-3 Fatty Acids for Managing DED

About 80% of respondents reported recommending omega-3 fatty acids, in either diet or supplement form, for DED management. This proportion is comparable to previously reported values of 70% of Australian optometrists [55] and 80% of New Zealand optometrists in surveys of DED practice patterns [56]. In the present study, we found more respondents reported recommending omega-3 fatty acids for DED management than for AMD. This is perhaps not surprising given that the anti-inflammatory effects of omega-3 fatty acids are widely promoted, and several clinical trials have demonstrated favorable results supporting the use of omega-3 supplements for improving clinical outcomes in DED [25]. 

Although the pathogenesis of DED is unclear, tear instability, tear hyperosmolarity, and neuro-sensory abnormalities are understood to contribute to a vicious cycle of inflammation that perpetuates on the ocular surface [16]. The primary etiological factor is considered to differ between the two main DED subtypes: the evaporative form of DED, which is frequently caused by meibomian gland dysfunction, and aqueous-deficient DED, which is often associated with aging and systemic autoimmune conditions [15]. Almost all the practitioners who reported recommending omega-3 fatty acids for DED indicated doing so for managing evaporative or mixed DED, rather than for aqueous-deficient DED. The rationale for this practice may relate to the potential for systemic fatty acid consumption to modify lipid production and regulate the lipid-secreting meibomian glands, both of which may be beneficial in evaporative DED [57,58]. However, it may be advisable to exercise caution in interpreting the result for aqueous-deficient DED, on account of the relative rarity of this subtype in an unmixed form, without an overlay of the more prevalent evaporative form [56]. Notwithstanding these practice patterns, the anti-inflammatory benefits conferred by omega-3 fatty acids are not restricted to improving tear-lipid dysfunction, as aqueous-deficient DED may be associated with an immune-mediated inflammatory response [16]. Notably, a recent Cochrane systematic review found that in DED, long-chain omega-3 supplements, compared to placebo, increased aqueous tear production and improved tear osmolarity, both of which are compromised in aqueous-deficient DED [25]. 

Our study established that practitioners are less likely to report recommending omega-3 fatty acids for milder forms of DED. This trend has been reported in previous evaluations of self-reported practices relating to recommending omega-3 fatty acids for DED, by eye care practitioners in New Zealand, Australia, and the UK [55,56]. It is unclear why this practice behavior exists given a lack of specific evidence to support this approach. In the DED staged management and therapy algorithm published in the 2017 Tear Film and Ocular Surface Society (TFOS) International Dry Eye Workshop II report (TFOS DEWS II), education regarding potential dietary modification (including oral essential fatty acid supplementation) is listed as a treatment approach for all DED stages, and not limited to only moderate or severe disease [59]. Whether perceived cost implications for patients at an early disease stage plays a role in this decision-making is not known.

Respondents indicated recommending a variety of daily omega-3 fatty acid dosages for managing DED, ranging from 250 mg to 6000 mg per day (median [IQR]: 2000 [1000–2750] mg/day). Published guidelines exist with regard to the optimal dose of omega-3 fatty acids for several other health conditions. For example, the American Heart Association recommends 4000 mg EPA, or combined EPA and DHA, per day for reducing systemic triglycerides in cases of hypercholesterolemia [60]. The International Society for Nutritional Psychiatry Research recommends 1000 to 2000 mg of EPA per day for treating major depressive disorders [61]. There are currently no formal recommendations for the optimal supplement dosage that may provide clinical benefit in ocular conditions. 

Randomized controlled trials (RCTs), comparing the efficacy of long-chain omega-3 fatty acids relative to placebo or to no intervention for treating DED, have adopted a wide range of dosages, varying across a 20-fold range, from 135 mg to 3000 mg of combined EPA and DHA per day [25]. This large dosing range could contribute to the apparently contrasting outcomes observed in different intervention trials [20,21,23]. Factors that may contribute to heterogeneity in the reported effect estimates of omega-3 supplement clinical efficacy for treating DED include: differences in study design, population selection, and the choice of outcome measures. There is also lack of certainty in the literature with regard to the optimal form of the long-chain omega-3 fatty acid supplements, which exist in triglyceride, ethyl ester, or phospholipid forms, and the optimal ratio of EPA to DHA for DED treatment [25]. Compounding these issues is the current lack of consensus on the most appropriate omega-3 fatty acid-prescribing protocol, specifically as related to dose, treatment duration, and composition, to achieve the most clinical benefit [59]. Of specific relevance to DED, there is emerging evidence to suggest that topical omega-3 fatty acid supplementation also may have a role in modulating ocular surface inflammation [62,63].

The two commercially available formulations reported to be most frequently recommended by survey respondents were Thera Tears™ (dosage recommended by manufacturer: three capsules daily, each containing 450 mg EPA, 300 mg DHA, 1337.5 mg ALA, and 183 IU vitamin E as an antioxidant) and Lacritec™ (dosage recommended by manufacturer: three capsules daily for six weeks, and two capsules daily thereafter, each capsule containing 134 mg EPA, 66.8 mg DHA, 58.5 mg oleic acid, 58.5 mg linoleic acid (LA), 192 mg linolenic acid, 434 mg Borago officinalis seed oil fixed, and 93.5 mg gamma-linolenic (GLA) acid). In addition to omega-3 fatty acids, Lacritec contains short-chain omega-6 fatty acids. Approximately one in five optometrists reported recommending combined omega-3 and omega-6 supplements for managing DED. While eicosanoids derived from the metabolism of the long-chain omega-6 fatty acid, AA, are pro-inflammatory, dietary intake of the short-chain omega-6, GLA, and its precursor, LA, can yield anti-inflammatory effects by acting as precursors to the eicosanoid prostaglandin-E1 (PGE1) [64]. The potential effects of omega-6 fatty acids on ocular surface inflammation are, therefore, complex.

The US Women’s Health Study found an association between a higher ratio of dietary omega-6 to omega-3 and an elevated risk of DED [19]. A recent cross-sectional study reported that relatively high omega-3 consumption and moderate omega-6 consumption were protective against meibomian gland dysfunction in postmenopausal women [65]. However, several RCTs assessing the efficacy of GLA and LA for alleviating ocular surface inflammation have reported inconsistent effects [25,66,67]. A recent Cochrane systematic review found that relative to placebo, combined omega-3 and omega-6 fatty acid supplements had no benefit on aqueous tear production or ocular surface staining. Although the pooled effect estimate indicated a possible improvement in tear stability with this intervention, the change was not considered clinically meaningful [25]. 

Baseline dietary omega-3 fatty acid intake is another important consideration for identifying patient populations likely to benefit from enhancing their omega-3 fatty acid intake. Supplementation in individuals already achieving sufficient essential fatty acid levels through their diet would not be expected to demonstrate the same biological response as in those who are deficient [68]. While 99% of surveyed optometrists in this study reported offering dietary advice to their patients, almost none used quantitative tools to survey their patients’ existing dietary habits. Techniques that can be used to estimate omega-3 intake include fatty acid assays from blood samples, although testing tends to be invasive, relatively costly and may not be readily accessible to all clinicians, and collection of short-term dietary records, which can be time consuming and, thus, not ideally suited for use in clinical settings. To overcome these barriers, simple food frequency tools may be of value for providing a rapid, non-invasive estimation of an individual’s omega-3 intake, and for informing clinical recommendations regarding the potential benefit, or otherwise, of supplementation or dietary modification [69].

### 4.4. Knowledge of the Potential Benefits and Risks of Omega-3 Fatty Acids

It has been estimated that in Australia, 80% of adults may not be meeting the suggested dietary intake of long-chain omega-3 fatty acids [9], which, as recommended by the National Health and Medical Research Council (NHMRC) is 430 mg per day for female adults and 610 mg per day for male adults [70]. Almost half (46%) of practitioners surveyed recommended for their patients to consume between two and four servings of fish or non-fish seafood per week to improve ocular health. However, less than one in 10 respondents estimated the closest to ideal approximate adult dietary target of marine-based omega-3 fatty acids as 500 mg/day (which approximates to consuming two servings of oily fish per week). Furthermore, less than one-third of respondents were aware that short-chain omega-3 fatty acids, obtained from plant-based sources, are less directly biologically active than long-chain omega-3 fatty acids [71,72]. This disparity highlights a potential target area for professional education to raise awareness about the relative biological efficacy of different food sources. 

High-dose omega-3 fatty acids, >2000 mg per day, tend to be safe and well tolerated but are not without potential adverse effects. The most common side effects are gastrointestinal (e.g., nausea, bloating), dermatological (e.g., skin itchiness), and hematological (e.g., anticoagulatory effects). Several studies examining the safety profile of omega-3 fatty acids, dosed at up to 4000 mg per day, have concluded that supplementation is generally well tolerated and that associated adverse events are unlikely to be of clinical significance [60,61,73,74,75]. Over half of survey respondents (53%) reported taking the safety profile of omega-3 fatty acids (i.e., side effects and contraindications) into consideration when making clinical recommendations. Optometrists who declared a clinical interest in DED or ocular surface disease were twice as likely to recommend omega-3 fatty acids to their patients (95% CI: 1.05–5.2) than those without such interests. Interestingly, respondents who recognized that omega-3 fatty acids have potential side effects were approximately four times more likely to recommend their use. While this might initially seem counterintuitive, it may be a testament to optometrists being cautious prescribers, and a likelihood that clinicians with greater awareness about the safety profile of omega-3 fatty acids are also more likely to be knowledgeable about their benefits. More extensive knowledge may bring about greater confidence in making clinical dietary recommendations. This is corroborated by the finding that survey respondents who declared an interest in DED or ocular surface disease were three times more likely to successfully demonstrate their knowledge about the underlying biological effects and dietary targets of omega-3 fatty acids than those who failed to correctly answer 60% or more of the relevant knowledge-focused questions. 

### 4.5. Sources of Information and Evidence Used to Guide Clinical Decision Making 

Four in five respondents selected continuing education conferences and/or articles as the main source of information used to guide their clinical decision making about omega-3 fatty acids. Two in five respondents indicated that they source primary research articles and/or systematic reviews for advice. 

The importance of evidence-based practice is well recognized in both optometric training and practice [76]. A previous study reported that while attitudes towards evidence-based practice were generally positive among eye care practitioners, a key barrier was a lack of clinician time to access and appraise large volumes of research evidence [76]. Specific barriers that appear to challenge the adoption of best practice relating to nutrition and dietary recommendations in practice include the apparently conflicting results from different studies, a perception from some clinicians that nutritional advice is outside the optometric scope of practice, and a paucity of available clinical tools for quantifying diet in clinical practice. 

A number of recent initiatives have sought to support optometrists in making evidence-based dietary recommendations that are relevant to eye health. Optometry Australia’s 2019 AMD Clinical Practice Guide recommends that optometrists inform patients about how AMD risk can be modified by diet and other lifestyle factors, such as smoking [77]. With respect to DED, the TFOS DEWS II reports provide a peer-reviewed, open-access summary of the current scientific evidence, based on expert consensus [78]. In the TFOS DEWS II report, providing patient education about potential dietary modifications (including oral omega-3 supplementation) is listed within the first step of the four-step staged management algorithm for DED. 

The current study identified current clinical practice patterns in primary eye care, as related to oral omega-3 fatty acids, and highlighted knowledge gaps and perceived barriers to clinical implementation. There were some limitations to the present study. While the cohort represented a broad range of optometric practice locations and modalities, there was the potential for self-selection bias, and this was reflected in the proportion of participants declaring a clinical interest in DED or ocular surface disease. Also noted was a relative over-representation of younger optometrists, and optometrists working in independent (private) practice. These limitations may affect the generalizability of our findings, but are not uncommon considerations in survey-based research.

## 5. Conclusions

Our results suggest that most Australian and New Zealand optometrists routinely provide clinical recommendations about diet and nutritional strategies, as relevant to eye health. While many optometrists indicated recommending diet-based strategies for managing AMD, the potential benefits of whole-food omega-3 sources relative to the limited benefits of omega-3 supplements were not consistently appreciated. Most respondents recommended omega-3 fatty acids for managing DED, despite conflicting data from recent randomized trials. Respondents were more likely to recommend omega-3 fatty acids to patients with moderate or severe (rather than mild) DED, and to use omega-3 supplementation to treat predominantly evaporative DED. Further research is needed to address uncertainties in the evidence relating to the optimal prescribing regimen (i.e., omega-3 dose and formulation composition) for treating DED. This information will be of value for informing future education programs about eye health and nutrition, particularly at events such as clinical education conferences that were reported to form the main source of information used by optometrists to guide clinical decision-making.

## Figures and Tables

**Table 1:** Summary of survey questions.

Section	Question Surveyed
**1. Practitioner demographics**	Gender Age Location of optometry degree completion Year of graduation from optometry degree Location of principal practice Postcode of principal practice Therapeutic endorsement status Mode of optometry practice (academic, corporate, hospital clinic or public health clinic, independent, refractive surgery clinic, other) Average hours per week providing patient care Approximate age distribution of patients (percentage for each of: under 18 years, 18–40 years, 41–60 years, over 60 years, so that percentages summed to 100%) Areas of self-declared clinical interest (contact lenses, diabetes, dry eye or ocular surface disease, gerontology or aged care, glaucoma, myopia, paediatrics or binocular vision, retinal disease, other)
**2. Self-reported clinical practices relating to diet and nutritional supplementation**	Frequency of enquiry about patients’ diet (never, rarely, sometimes, often or always) Whether quantitative tools were used to survey dietary habits * Frequency of enquiry about patients’ usual intake of nutritional/vitamin supplements (never, rarely, sometimes, often or always) Frequency of providing dietary advice to patients (never, rarely, sometimes, often or always) Statement that best describes respondents’ current general clinical approach to omega-3 fatty acid recommendations for ocular health ^^^ Sources of information used to guide clinical decision-making regarding omega-3 fatty acids Percentage of patients seen in practice estimated to have AMD Percentage of patients seen in practice estimated to have DED
**3a. Clinical approach to recommending omega-3 fatty acids for AMD and DED**	**Section 3a ^#^**
	**Age-related macular degeneration (AMD)** Frequency of recommending foods rich in omega-3 fatty acids Specific food sources recommended (fish, non-fish seafood [e.g., prawns, oysters etc.], cooking oils or margarines, vegetables or fruits, nuts and seeds, other) * If fish and/or seafood was selected above: frequency of intake recommended Frequency of recommending plant-based omega-3 supplements Frequency of recommending marine-based omega-3 supplements Open text box to record specific recommendations relating to omega-3 supplements for AMD **Dry eye disease (DED)** Frequency of recommending foods rich in omega-3 fatty acids Specific food sources recommended (fish, non-fish seafood [e.g., prawns, oysters etc.], cooking oils or margarines, vegetables or fruits, nuts and seeds, other) * If fish and/or seafood was selected above: frequency of intake recommended Frequency of recommending any omega-3 supplements Specific recommendations relating to the type of supplement(s) * Open text box to record specific recommendations relating to omega-3 supplements for DED Selection of DED severity considered for recommending omega-3 fatty acids via dietary modification or supplementation Selection of DED disease subtypes considered for recommending omega-3 fatty acids via dietary modification or supplementation
**3b. Reason(s) for not making omega-3 recommendations or for recommending that patients not consume omega-3 fatty acids**	**Section 3b ^#^**
	Reason(s) for not currently recommending omega-3 fatty acids (from either the diet or via supplementation) If previously recommended omega-3 fatty acids, reason(s) for no longer making this recommendation Other dietary supplements recommended to patients
**4. Knowledge of omega-3 fatty acids**	Awareness of differences in the biological effects of plant-based and marine-based omega-3 fatty acids Perceived anti-/pro-inflammatory effects of omega-3 fatty acids Perceived anti-/pro-inflammatory effects of omega-6 fatty acids Perceived ideal ratio of omega-6:omega-3 in dietary intake Knowledge of the approximate recommended adult daily dietary target of long-chain omega-3 fatty acids Yes/no choice about whether omega-3 fatty acids are considered to have any potential side effects If yes, perceived side effects associated with omega-3 fatty acids (options offered: nose bleeds, bloating, constipation, gastric reflux [fishy after-taste], hypercholesterolaemia [high systemic levels of cholesterol], excess lacrimation, other) Perceived daily safety limit for omega-3 fatty acid intake Perceived contraindications to omega-3 fatty acid supplementation

^^^ Stratification question, depending on the response selected, participants were directed to either Section 3a or Section 3b. ^#^ Only one of these sections was displayed to the respondent. * Only displayed if any answers other than “Never” were selected in the previous question. AMD, age-related macular degeneration; DED, dry eye disease.

**Table 2:** Summary of participant demographics.

Characteristic (*n* = 206 for All Categories)	Number of Responses (%)
**Gender**	
Male	80 (38.8)
Female	125 (60.7)
Other/gender diverse	1 (0.5)
**Age** (years)	
<30	91 (44.2)
31–45	67 (32.5)
46–60	39 (18.9)
>60	9 (4.4)
**Completed optometry training in Australia or New Zealand?**	
Yes	194 (94.2)
No	12 (5.8)
**Country of principal practice**	
Australia	131 (63.6)
New Zealand	75 (36.4)
**Therapeutically endorsed**	
Yes	180 (87.4)
No	26 (12.6)
**Principal type of optometric practice**	
Academic	16 (7.8)
Corporate	61 (29.6)
Hospital or public health clinic	22 (10.7)
Independent	101 (49.0)
Refractive surgery clinic	2 (1.0)
Other	4 (1.9)
**Average hours spent providing patient care as an optometrist per week**	
0	2 (1.0)
1–10	20 (9.7)
11–20	23 (11.2)
>20	161 (78.2)
**Areas of specific clinical interest**	
Binocular vision	58 (28.2)
Contact lenses	96 (46.6)
Diabetes	65 (31.6)
Dry eye or ocular surface disease	103 (50.0)
Gerontology or aged care	17 (8.3)
Glaucoma	72 (35.0)
Myopia	120 (58.3)
Paediatrics	58 (28.2)
Retinal disease	54 (26.2)
Other	12 (5.8)

**Table 3:** Factors predicting whether optometrists recommend omega-3 fatty acid consumption (from food sources or supplementation) to improve eye health.

Factor	Univariate Analysis		Multivariate Analysis	
	OR (95% CI)	*p* Value	OR (95% CI)	*p* Value
**Gender** (*n =* 206)				
Male	Ref			
Female	0.76 (0.38–1.54)	0.450		
**Age**, in years (*n =* 206)				
<30	Ref			
31–45	2.04 (0.92–4.49)	0.078		
>45	2.34 (0.93–5.89)	0.070		
**Optometric practice experience,** in years (*n =* 206)				
<10 years	Ref			
11–20	2.31 (0.82–6.49)	0.111		
21–30	2.10 (0.74–5.91)	0.162		
>31 years	1.63 (0.51–5.19)	0.412		
**Country of practice** (*n =* 206)				
Australia	Ref			
New Zealand	1.48 (0.72–3.04)	0.288		
**Therapeutically endorsed** (*n =* 206)				
No	Ref			
Yes	0.27 (0.06–1.21)	0.087		
**Principal type of optometric practice** (*n =* 206)				
Academic	Ref		Ref	
Corporate	1.84 (0.57–5.92)	0.306	1.87 (0.49–7.09)	0.356
Hospital or public health clinic	1.05 (0.28–3.99)	0.943	0.96 (0.22–4.30)	0.959
Independent	4.06 (1.26–13.06)	0.019	2.51 (0.67–9.38)	0.171
**Average weekly hours spent providing patient care as an optometrist** (*n =* 206)				
1–10	Ref			
11–20	0.90 (0.21–3.94)	0.889		
>20 hours	0.97 (0.30–3.10)	0.959		
**Self-declared clinical interest in DED or ocular surface disease** (*n =* 206)				
No	Ref			
Yes	4.58 (2.12–9.91)	<0.001	3.10 (1.31–7.36)	0.010
**Self-declared clinical interest in gerontology or aged care** (*n =* 206)				
No	Ref			
Yes	0.62 (0.21–1.88)	0.401		
**Self-declared clinical interest in retinal disease** (*n =* 206)				
No	Ref			
Yes	1.07 (0.51–2.27)	0.857		
**Pass mark (scoring ≥3 out of 5) on ‘omega-3 knowledge score’** (*n =* 191)				
No	Ref			
Yes	1.04 (0.37–2.99)	0.936		
**Recognised that omega-3 fatty acids have potential side effects** (*n =* 191)				
No	Ref		Ref	
Yes	3.94 (1.88–8.25)	<0.001	2.34 (1.05–5.22)	0.037

DED, dry eye disease; OR, odds ratio; Ref, reference.

**Table 4:** Predictive factors for a ‘pass’ mark on the omega-3 knowledge score, relating to the accuracy of practitioners’ general knowledge about omega-3 fatty acids.

Factors (*n* = 206 for All Categories)	Univariate Analysis		Multivariate Analysis	
	OR (95% CI)	*p* Value	OR (95% CI)	*p* Value
**Gender**				
Male	Ref			
Female	1.35 (0.55–3.34)	0.511		
**Age** (years)				
≤30	Ref			
31–45	1.93 (0.68–5.50)	0.219		
>45	2.41 (0.81–7.15)	0.113		
**Optometric practice experience** (years)				
≤10	Ref			
11–20	1.49 (0.48–4.64)	0.492		
21–30	1.66 (0.53–5.21)	0.384		
>31	1.52 (0.40–6.04)	0.549		
**Country of practice**				
Australia	Ref			
New Zealand	2.04 (0.86–4.83)	0.106		
**Therapeutically endorsed**				
No	Ref			
Yes	0.55 (0.18–1.62)	0.276		
**Principal type of optometric practice**				
Academic	Ref			
Corporate	0.07 (0.01–0.76)	0.029	0.08 (0.01–0.86)	0.037
Hospital or public health clinic	1.25 (0.25–6.29)	0.787	1.68 (0.31–9.02)	0.544
Independent	0.77 (0.19–3.06)	0.710	0.72 (0.18–2.94)	0.645
**Average weekly hours spent providing patient care as an optometrist** (years)				
1–10	Ref			
11–20	0.85 (0.11–6.70)	0.877		
>20 h	1.24 (0.27–5.81)	0.783		
**Self-declared clinical interest in DED or ocular surface disease**				
No	Ref			
Yes	2.84 (1.12–7.20)	0.028	2.97 (1.09–8.12)	0.034
**Self-declared clinical interest in gerontology or aged care**				
No	Ref			
Yes	0.54 (0.14–2.08)	0.372		
**Self-declared clinical interest in retinal disease**				
No	Ref			
Yes	1.45 (0.51–4.10)	0.489		
**Omega-3 fatty acids (from diet or supplementation) recommended in their practice for improving ocular health**				
No	Ref			
Yes	1.04 (0.37–2.99)	0.936		

DED, dry eye disease; OR, odds ratio; Ref, reference.

## Appendix A

**Table A1:** Knowledge questions surveyed and, in bold, answers considered as ‘correct’ for deriving an ‘omega-3 knowledge score’.

Questions (Answer Considered ‘Correct’ in Bold)
There are two forms of omega-3 fatty acids, derived either from plant-based sources (e.g., chia seeds, walnuts, flaxseed) or marine-based foods (e.g., fish and seafood).
3.1 Which of the following do you consider to **best** describe the difference between plant-based and marine-based omega-3 fatty acids, if they are consumed at the same dose? (a) Plant-based (short-chain) omega-3 fatty acids have MORE biological effect than marine-based (long-chain) omega-3 fatty acids (b) **Plant-based (short-chain) omega-3 fatty acids have LESS biological effect than marine-based (long-chain) omega-3 fatty acids** (c) Plant-based (short-chain) omega-3 fatty acids have the SAME biological effect as marine-based (long-chain) omega-3 fatty acids (d) Don’t know
Omega-6s are the other main form of essential fatty acids. For the next two questions, consider which option **best** describes the biological roles of omega-3 and omega-6 fatty acids.
3.2 Omega-3 fatty acids are: (a) **Always anti-inflammatory** (b) Mostly anti-inflammatory (c) Equally anti-inflammatory and pro-inflammatory (d) Mostly pro-inflammatory (e) Always pro-inflammatory
3.3 Omega-6 fatty acids are: (a) Always anti-inflammatory (b) Mostly anti-inflammatory (c) Equally anti-inflammatory and pro-inflammatory (d) **Mostly pro-inflammatory** (e) Always pro-inflammatory
3.4 Based upon your current knowledge, what do you consider to be an ideal ratio of omega-6 to omega-3 in the human diet? (a) 10:1 (b) **4:1** (c) 1:1 (d) 1:4 (e) 1:10
3.5 Based on your understanding, which of the following options would you consider to be closest to the ideal approximate adult dietary target of long-chain (marine-based) omega-3 fatty acids (from food and/or supplementation)? (a) 100 mg/day (b) 250 mg/day (c) **500 mg/day** (d) 1000 mg/day (e) 1500 mg/day
